# The Misuse of Medical Charities

**Published:** 1906-12-15

**Authors:** 


### The Hospital

A Journal of

The Medical Sciences and Hospital Administration.

Vol. XLI.—No. 1,056. SATURDAY, DECEMBER 15, 1906.

### The Misuse of Medical Charities.

Those who read the report of the Conference of Representatives of Hospitals and of the Medical Profession throughout the United Kingdom, as published in another column, will find abundant evidence of the excessive free medical relief granted by the hospitals in this country under the existing system. It is essential that measures shall be taken to reorganise existing methods, for such a reorganisation has become a matter of urgent necessity, equity and probity. It is a necessity because the very existence of the voluntary principle of hospital support, the well-being and maintenance of an adequate number of general practitioners throughout the country, and in consequence the welfare of all classes of the community, are at stake. It is equitable that a revolution in system and method should take place, for the present system yields injustice to the hospitals and their medical officers, to the great body of medical practitioners, to the supporters of the hospitals, and to the patients who most urgently need medical aid. All this was demonstrated by the speakers at the meeting in Hanover Square on the 3rd instant, and at the Conference on the 6th instant. It is impossible to study the subject and to doubt for one moment that this great question of medical relief must be reconsidered in every aspect as a matter of justice to all the interests concerned.

None can be more impressed with the difficulties which have to be encountered than we are. A great question like that under consideration must be faced with courage, energy, and persistence. It will require the combined efforts of all the most disinterested and able men who are at present responsible for the administration of hospitals, the treatment of their patients, and the protection of the just claims of the great body of medical practitioners. All pretence and make-believe, all narrcwness of view and self-interest, must yield to the cardinal fact, that, the existing sytem has broken down in practice, and must give place to one, which, whilst it will leave free medical relief readily accessible to all who are entitled to it, will at the same time tend to stiffen the moral fibre of the people and protect the just claims of all the interests involved. That is why we have urged that this is a matter of probity, for any new and complete scheme, if it is to prove effective, must rest upon the highest principles, and must display wisdom and knowledge in its construction. Time, therefore, will be required for adquate discussion and conference, before any such plan can be successfully evolved and brought into operation.

The proceedings at the Conference will be read with interest, and show that this view prevailed. The Conference was widely representative of the many interests involved; it was largly attended; those present were genuinely interested; but the feeling exists, that, too little progress was made. We do not share this feeling, however, owing to the considerations we have just laid down. It has its origin, probably, in the circumstance that instead of the main resolution, which was unanimously adopted, approving generally of the principles, it merely welcomed their further consideration as set forth in the proposals of the Joint Hospitals Committee. Without much discussion, or any previous clearing of the ground by correspondence with those who lacked full knowledge and so were not in agreement, a general approval by the meeting of all these principles might have caused justifiable dissatisfaction. As matters stand, however, a *modus vivendi* has been found which permits all the interests represented at the Conference, to thresh out the whole question with the standing committee of the Conference. We cannot forget that the proposals of the Conjoint Hospitals Committee were twenty-eight in number. They covered a very wide field, and it was manifestly impossible for a large public meeting to discuss them in detail. The great point to secure was an organisation, to work away at the problems presented, and to bring all these matters into concrete form by communication with the representatives of the interests represented, so as to make it possible for the adoption, by another Conference, of a perfected scheme calculated to remedy the evils complained of. This great step in advance has been taken. An annual Conference will now be held, and a representative standing committee, acting in conjunction with the Hospitals Committee of the British Medical Association, has been appointed. If the Committee does its work properly, a practical scheme should be forthcoming in the course of the next few months. In these circumstances, everybody interested in the subject has good cause to be satisfied with the steps taken in the direction of removing the existing abuses, and establishing a comprehensive system of medical service throughout the country. Such a system, if it safeguards the interests concerned, should prove, in practice, a boon to all classes of the community.

